# Blast resistance in Indian rice landraces: Genetic dissection by gene specific markers

**DOI:** 10.1371/journal.pone.0211061

**Published:** 2019-01-23

**Authors:** Manoj Kumar Yadav, S. Aravindan, Umakanta Ngangkham, S. Raghu, S. R. Prabhukarthikeyan, U. Keerthana, B. C. Marndi, Totan Adak, Susmita Munda, Rupesh Deshmukh, D. Pramesh, Sanghamitra Samantaray, P. C. Rath

**Affiliations:** 1 ICAR-National Rice Research Institute, Odisha, India; 2 National Agri-Food Biotechnology Institute (NABI), Mohali Punjab, India; 3 Rice Pathology Laboratory, AICRIP, Gangavathi, University of Agricultural Sciences, Raichur, India; ICAR-Indian Institute of Rice Research, INDIA

## Abstract

Understanding of genetic diversity is important to explore existing gene in any crop breeding program. Most of the diversity preserved in the landraces which are well–known reservoirs of important traits for biotic and abiotic stresses. In the present study, the genetic diversity at twenty-four most significant blast resistance gene loci using twenty-eight gene specific markers were investigated in landraces originated from nine diverse rice ecologies of India. Based on phenotypic evaluation, landraces were classified into three distinct groups: highly resistant (21), moderately resistant (70) and susceptible (70). The landraces harbour a range of five to nineteen genes representing blast resistance allele with the frequency varied from 4.96% to 100%. The cluster analysis grouped entire 161 landraces into two major groups. Population structure along with other parameters was also analyzed to understand the evolution of blast resistance gene in rice. The population structure analysis and principal coordinate analysis classified the landraces into two sub–populations. Analysis of molecular variance showed maximum (93%) diversity within the population and least (7%) between populations. Five markers viz; K3957, Pikh, Pi2–i, RM212and RM302 were strongly associated with blast disease with the phenotypic variance of 1.4% to 7.6%. These resistant landraces will serve as a valuable genetic resource for future genomic studies, host–pathogen interaction, identification of novel *R* genes and rice improvement strategies.

## Introduction

Rice (*Oryza sativa* L.) is the staple food for more than half of the world’s population [1] and depends on rice for more than 20% of their daily calorie intake [2]. By 2035, it is expected that an extra 116 million tonnes of rice will be required to feed the world’s increasing population [3]. This projected production has to be inevitably met with the expected water scarcity, less arable land, new emerging pathogens and pests and likely adverse effects of climate change [4].

The rice crop is affected by several diseases, of which blast disease caused by the fungus *Magnaporthe oryzae* is one of the most devastating disease causing enormous losses worldwide. *M*. *oryzae* can infect rice plant right from seedling to late vegetative/reproductive stages affecting leaves, nodes, collar, panicles, panicle neck, and roots [5]. The fungal pathogen, *M*. *oryzae* has been placed among the top 10 fungal plant pathogens in the world based on its scientific and economic importance [6]. Owing to its presence and survival in different environmental conditions in more than 85 countries, it causes a yield loss that is enough to feed more than 60 million people each year [7]. Use of resistant cultivars, fungicides, optimum fertilizer applications and appropriate planting dates are some of the strategies to manage the disease [8]. Though fungicide application to control the disease is feasible, it remains economically unprofitable for resource poor farmers and an possesses environmental risk at high application rates [9]. The utilization of *R* (resistant) genes is the most economically viable and environmentally friendly choice for the control of this disease. Resistance is generally conferred by either major *R* genes that provide complete protection against few races of the pathogen or minor genes, which conferred partial protection [10].

To date, more than 100 *R* genes, and more than 350 QTLs for resistance to rice blast have been identified, and 27 have been molecularly cloned and characterized *viz*., *Pib*, *Pb1*, *Pita*, *Pi9*, *Pi2*, *Pizt*, *Pid2*, *Pi33*, *Pii*, *Pi36*, *Pi37*, *Pikm*, *Pit*, *Pi5*, *Pid3*, *Pid3–A4*, *Pikh*, *Pish*, *Pik*, *Pikp*, *Pia*, *PiCO39*, *Pi25*, *Pi1*, *pi21*, *Pi50* and *Pi65(t)* [11,12,13]. The rapid changes in virulence characteristics that take place in pathogen populations remains a constant challenge to the success of existing blast–resistant varieties of rice. However, the major blast resistance gene has been useful and should play a vital role in rice production if they are cautiously selected and deployed [14]. Hence, there is an imperative need for mining the new *R* genes/alleles and minor resistance genes in landraces in which resistance has co–evolved along with fungus over thousands of years. Genetically diverse rice landraces are one of the most important sources for major blast resistance to be introgressed into rice cultivars for the control of the blast [15]. Though breeding strategies focus on developing a durable and broad–spectrum resistant variety by pyramiding many *R* genes into popular rice cultivar through marker assisted selection [16,17], there is always need for a novel resistance allele to combat continuously evolving pathogen.

With the discovery and fine mapping of blast resistance genes, many PCR–based markers have been developed and employed in the mining of blast resistance genes in the diverse elite germplasm which contain untapped resources of discrete alleles. The potential of elite germplasm will remain unknown unless the efforts are initiated to screen them intended for their possible use and function [18,19,20]. Accurate identification of a specific *R* gene in diverse elite germplasm through DNA markers and differential blast races is an essential step in ensuring the accuracy in utilization of *R* gene in MAS for different rice breeding program [19]. The introduction of modern rice cultivars may lead to the erosion of genetic resources like landraces and traditional varieties from the farmer’s fields which resulted in the loss of genetic diversity of rice as well. However, the genetic diversity is to some extent maintained and preserved in the gene banks in the form of landraces/wild collections [21]. To date, seven blast epidemics have occurred from 1980 to 1987 in different states of India, *viz*. Andhra Pradesh, Himachal Pradesh, Haryana, and Tamil Nadu resulted in severe yield losses in the farmer’s fields [22] which necessitates in assessing the genetic diversity of the blast resistance genes in the landraces/germplasm. Since, the landraces has provided a rich source for genetic improvement of rice for specific traits and represent rich sources of specific allelic variation. India has a rich genetic diversity of landraces due to the diverse agro–climates and growing conditions, therefor, the present study, aimed to investigate a) the genetic diversity of major blast resistant genes b) identify donors for blast resistance and c) genetic association of markers with the blast resistance traditional in rice landraces collected from nine major rice growing states of India. The outcome of present study will help in identification of novel valuable genetic resource for rice blast resistance genes for development of durable blast resistant varieties in India.

## Material and methods

### Plant material

**Fig 1:**
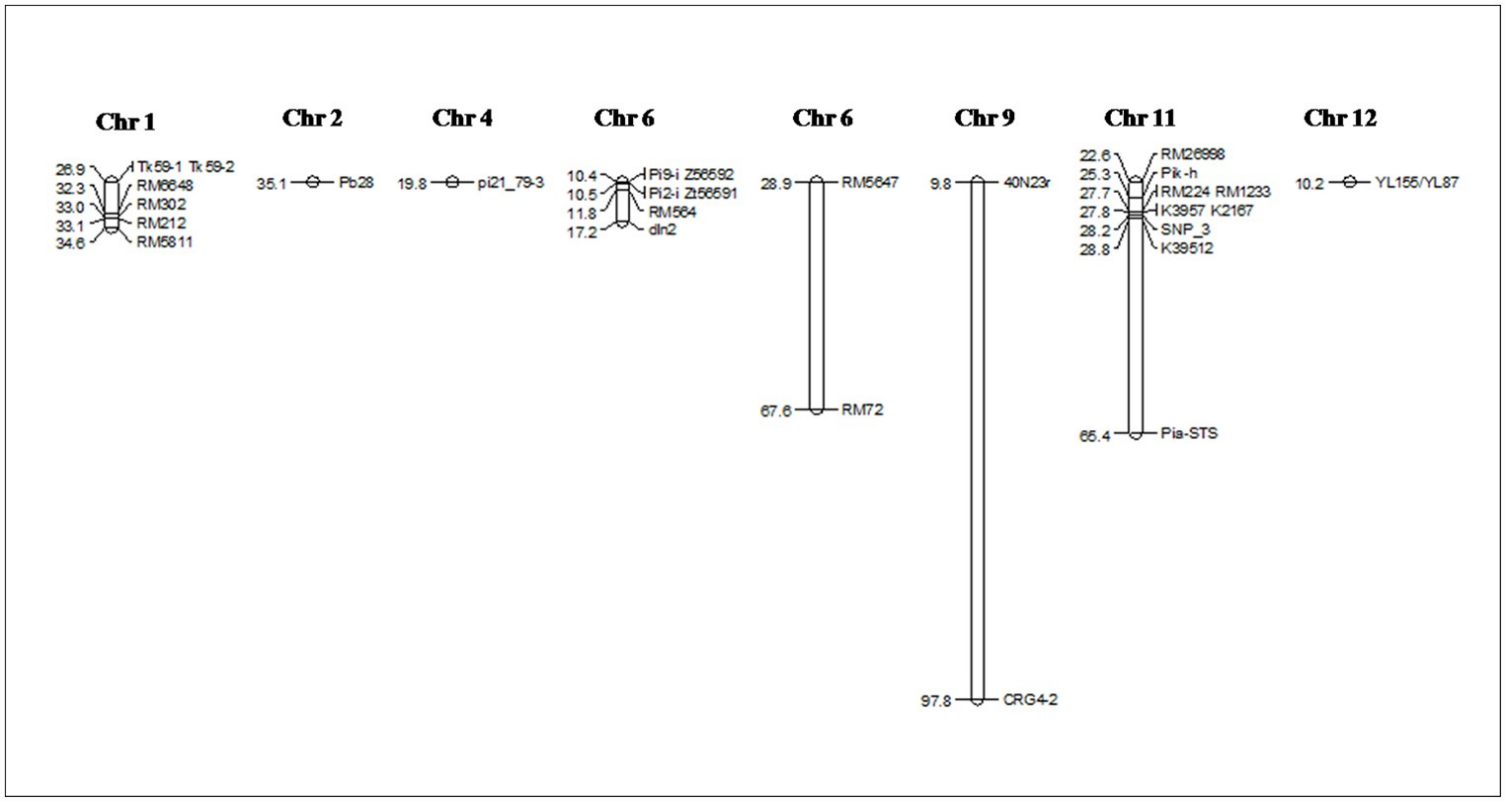
Diagrammatic representation of rice landraces belonged to different states of India.

A set of 161 diverse Indian rice landraces obtained from the National Gene Bank, ICAR–National Rice Research Institute, Cuttack were used in this study was. These landraces were collections of nine states of India with diverse ecologies (Fig 1). The details of 161 landraces are given in S1 Table.

### Disease reaction in uniform blast nursery

A total of 161 landraces from all over India, representing nine states was screened for their leaf blast resistance under natural condition in the Uniform Blast Nursery (UBN) at the experimental farm of ICAR–NRRI, Cuttack (85°55′48″ E longitudes and 20°26′35″ N latitude) as described in Yadav et al [9]. The leaf blast screening was conducted twice during two wet seasons of 2015 & 2016 with two replications. Each landrace (30 plants/test entry) was planted in 50 cm long rows in nursery beds at row spacing of 10 cm apart. In addition, the known susceptible checks (HR12 and CO39) were sown in borderlines as spreader rows as well as after every five test entries for the uniform spread of the disease. Disease reaction was recorded twenty five days after sowing and continued up to the 40^th^ day after sowing or the spreader row/checks achieved 85% of the disease symptom. Reactions of each landrace for leaf blast were scored as per the Standard Evaluation System (SES) of IRRI, (2002). The test entries with 0–3 scores were graded as highly resistant (HR), 4–5 as moderately resistant (MR), and 6–9 as susceptible (S). Whenever differences observed in score between the replications, the higher value was considered for scoring.

Location severity index (LSI) was calculated using following formula:

LSI=Score×Entries×100/Totalnumberofentries

### Genomic DNA extraction

Young leaf tissues of all the test entries were collected from two weeks old seedlings and stored at –80°C for genomic DNA extraction. Genomic DNA was isolated using the cetyltrimethyl ammonium bromide (CTAB) method [23] with slight modifications. The DNA quality and quantity were estimated based on 0.8% agarose gel electrophoresis and NanoDrop ND–1000 Spectrophotometer (Thermofisher scientific, USA). The Genomic DNA samples were finally diluted with 1X TE buffer to 20 ng/μl and stored at –20°C for further uses.

### Genotyping of rice blast *R* genes

**Fig 2:**
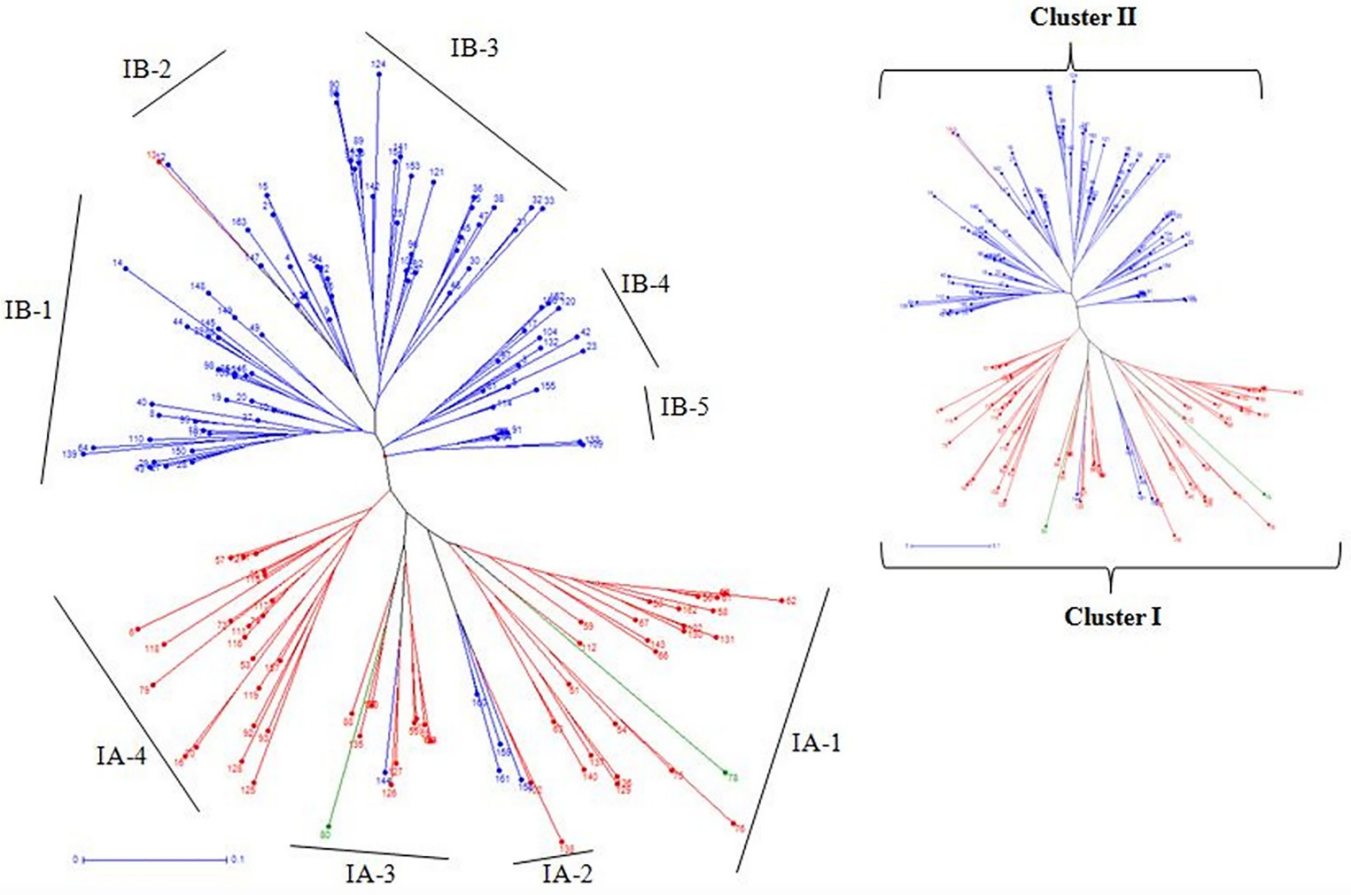
Graphical representation of blast *R* genes distribution on chromosomes showing the physical location. The names of the markers are given on the right side and the physical positions on the left side of the map.

The entire set of 161 landraces were genotyped using markers specific to 24 different blast resistance genes viz. *Pib*, *Pb1*, *Pita*, *Pi9*, *Pi2*, *Pizt*, *Pid2*, *Pi33*, *Pi36*, *Pi37*, *Pikm*, *Pit*, *Pi5*, *Pi54*, *Pish*, *Pik*, *Pikp*, *Pia*, *Pi25*, *Pi1*, *pi21*, *Pi56*, *Pi65(t)* and *Piz*. A total of twenty eight functional/linked markers corresponding to the twenty four *R* genes were used for screening of the blast resistance genes. The details of the markers used in the present study are shown in Table 1 with the physical locations on the corresponding chromosomes in Fig 2.

**Table 1:** List of markers used for blast resistance and their details.

S.N.	Gene	Marker used	Forward (5`-3`)	Reverse (5`-3`)	Type of marker*	Reference
1	*Pib*	Pb28	gactcggtcgaccaattcgcc	atcaggccaggccagatttg	SNP	[28]
2	*Piz*	Z56592	ggacccgcgttttccacgtgtaa	aggaatctattgctaagcatgac	SNP	[28]
3	*Piz-t*	Zt56591	ttgctgagccattgttaaaca	atctcttcatatatatgaaggccac	SNP	[28]
4	*Pik*	K39512	gccacatcaatggctacaacgtt	ccagaatttacaggctctgg	SNP	[28]
5	*Pik-p*	K3957	atagttgaatgtatggaatggaat	ctgcgccaagcaataaagtc	SNP	[28]
6	*Pikm*	k2167	cgtgctgtcgcctgaatctg	cacgaacaagagtgtgtcgg	InDel	[51]
7	*Pikh*	Pikh	caatctccaaagttttcagg	gcttcaatcactgctagacc	FM	[33]
8	*Pi9*	Pi9-i	gctgtgctccaaatgaggat	gcgatctcacatcctttgct	FM	[36]
9	*Pi2*	Pi2-i	cagcgatggtatgagcacaa	cgttcctatactgccacatcg	FM	[36]
10	*Pita/Pita2*	YL155/YL87	agcaggttataagctaggcc	ctaccaacaagttcatcaaa	FM	[29,30]
11	*Pi5*	40N23R	tgtgaggcaacaatgcctattgcg	ctatgagttcactatgtggaggct	InDel	[52]
12	*Pit*	tk59-1	atgataacctcatcctcaataagt	gttggagctacggttgttcag	FM	[32]
	*Pit*	tk59-2	atgataacctcatcctcaataagt	ccaagggattaggtcctagtg	FM	[32]
13	*Pid2*	dln2	gcgtcgaagatgtcctgaagctca	ggcagtcgtattgctgtgaa	FM	[53]
14	*Pish*	RM6648	gatcgatcatggccagagag	acagcaggttgatgaggacc	LM	[54]
		RM5811	ttcgcgctctccaagctc	ggatttggtcgaacaggttg	LM	[54]
15	*Pb1*	RM26998	acgcacgcacatcctcttcc	cggttctccatctgaaatccctagc	LM	[32]
16	*Pi33*	RM72	ccggcgataaaacaatgag	gcatcggtcctaactaaggg	LM	[55]
17	*Pia*	Pia-STS	cttttgagcttgattggtctgc	ctattgcaccagagggaccag	FM	[56]
18	*Pi1*	RM1233	gtgtaaatcatgggcacgtg	agattggctcctgaagaagg	SSR	[57]
		RM224	atcgatcgatcttcacgagg	tgctataaaaggcattcggg	SSR	[58]
19	*pi21*	pi21_79–3	gatcctcatcgtcgacgtctggc	agggtacggcaccagcttg	InDel	[59]
20	*Pi56*	CRG4-2	cctgtcagtctttccgagag	gaatccggtagctcaaggtg	Gene specific	[60]
21	*Pi65*	SNP_3	tgccaccagccatcttcaacat	accacatcactcatcgccatcc	InDel	[12]
22	*Pi36*	RM5647	actccgactgcagtttttgc	aacttggtcgtggacagtgc	SSR	[61]
23	*Pi37*	RM302	tcatgtcatctaccatcacac	atggagaagatggaatacttgc	SSR	[62]
		RM212	ccactttcagctactaccag	cacccatttgtctctcattatg	SSR	[62]
24	*Pi25*	RM564	catggccttgtgtatgcatc	atgcagaggattggcttgag	SSR	[63]

*SNP- Single nucleotide polymorphism, InDel- Insertion Deletion, FM- Functional marker, LM- Linked marker, SSR- Simple sequence repeats

### PCR amplification

Polymerase chain reaction was carried out in a 25 μl reaction volume with the following composition: 20 ng of genomic DNA, 0.2 μM of dNTP (25 mM), 0.2 μM of primers, 1.5 mM of MgCl_2_, 1X *Taq* buffer and 1U of *Taq* DNA polymerase (Thermo Scientific, USA). The PCR program was conducted as: initial denaturation at 94°C for 5 min; followed by 35 cycles of denaturation for 30 sec at 94°C, primers annealing for 30 sec at different temperatures (Table 1), and elongation at 72°C for 1 min, with 10 min final elongation at 72°C. The PCR amplified products were separated in 2.5–3% agarose gels along with a 100 bp DNA ladder (BR Biochem Life Sciences, India) and visualized through staining with ethidium bromide. The gel pictures were taken under UV light in a gel documentation system (AlphaImager, USA). All PCR reactions were repeated twice to confirm the results.

### Allele scoring and diversity analysis

The amplified PCR products of 28 markers were scored based on the presence (1) or absence (0) to create binary matrix for each marker. The genetic distance and similarity coefficients were estimated using the binary matrix of 28 markers. Major allele frequency, gene diversity and Polymorphism information content (PIC) value of each marker were estimated using Powermarker program Ver3.25 software [24]. An unweighted Neighbor Joining tree was constructed by the calculated NEI coefficient of dissimilarity index [25] using DARwin5 software [26].

### Association analysis

The hypothesis of genetic association between blast resistance genes and blast disease was tested through a general linear model (GLM) function using TASSEL5 software [27]. The TASSEL 5 software was run with one thousand permutations of data and it represents a significant association only if the *P*–value was observed in <5% of the permutations for the most significant polymorphism in a region.

### Population structure

Population structure analysis of 161 landraces based on 28 markers was investigated using STRUCTURE version 2.3.4 [28]. The number of subpopulations (K) was estimated using the programme at different K value by setting at K = 1 to 10, with 5 independent iterations per K using the admixture model and allele frequencies correlated. Each run, was based on 200,000 Markov chain Monte Carlo (MCMC) iterations after 200 000 burn–in phase. The peak value of ΔK was estimated to determine the optimal K as explained by Evanno et al [29] using the STRUCTURE HARVESTER programme [30]. The binary data matrix of twenty eight markers was used to develop pairwise individual genetic distance, to compute the PCoA (Principal Coordinate Analysis) in GenAlEx 6.502 [31]. All the other genetic analyses such as Analysis of Molecular Variance (AMOVA) and pairwise F_ST_ were performed using the GenAlEx version 5.0 software [31].

## Results

### Disease reactions of landraces

Based on the blast disease scoring data for consecutive two seasons in the UBN, 161 landraces were categorized into three groups; twenty one (HR; 13.04%) were highly resistant (score 0–3), seventy (MR; 43.47%) exhibited moderate resistance (score 4–5) and seventy (S; 43.47%) showed susceptible reaction (score 6–9) (Table 2). The location severity index (LSI) of the two seasons was calculated to know the disease reaction of landraces and it was found to be 5.04. A total of 91 landraces (56.52%) showed resistant (HR and MR) against the blast disease while 70 landraces were found susceptible (S) to disease reaction (43.47%) (S1 Fig). Out of the 21 HR landraces, the maximum number of HR landraces was the collections of Sikkim (7) followed by Maharashtra (5), Tripura (4), Gujarat (4) and Punjab (1) (Table 2). It shows that more than 50% landraces showing HR (11) were the collections of North–Eastern states of India (Sikkim and Tripura).

**Table 2:** Disease reactions of all India landraces for leaf blast resistance in the uniform blast nursery.

S. No.	Disease reaction	State	No. of resistant lines	Common name
1	Resistant	Punjab (1), Sikkim (7), Maharashtra (5), Tripura (4), Gujarat (4)	21	CM 76, Champe, Champei Sali, Chirakhey, Bada Atte, Lamo, Khimti, Jhapaca, Karjat-2, RP, Akola, Jyoti, Jaya, Garomalati, Khasa, Upahar, Birun, Jaya Gujari, Jeeram, Dadri Kalam (s), and Dangir (s)
2	M-Resistant	Maharashtra (1), Tamil Nadu (3), Kerala (2), Orissa (3), Punjab (1), Sikkim (10), Maharashtra (9), Tripura (19), and Gujarat (22)	70	Dodgyab 2–2, Mypali, Bangarutheega, Pormbalai, Velluliari kayama, Punsana No. 83, Usa, DI 3, Mypali, Basumati 370, Anadi, Basmati (s), Taichung, Kanchu ate, Baghey tulashi (s), Chhota Atte, Atte, Atte, Thima, Kal Chanti(s), Karjat-3, Kankuri, Basumati, Karjat-14, Kolpi, Saurav, Walle, Gouti, Chitalgam, Paijam, Bini (s), Ranjit, Kali kasa Garu (s), Karn rung, Kain chali, Kampai, Kapro, Paijam(chhota), Gabbori, Biran, Bini(s), Khasa(Black), Kartik Sal, Khasa(White), Benni, Khasa(Black), Binni Red, Gujarat-70, Sandal Basmati (s), Sonam (s), Jaad, Kamod Krishna (s), Gujrati Mhsuri, Saket, Central Basmati (s), Tata, Poigra, Moti, Gujarat-70, Kada, Dangi, Mahsuri-1, Ghode, Ambamohar (s), Moti Sathi, GR-101 (s), GR-103, Narmada and Kulam
3	Susceptible	Madhya Pradesh (1), Tamil Nadu (8), Kerala (3), Odisha (1), Sikkim (5), Maharashtra (16), Tripura (17), and Gujarat (19)	70	Chinoot, Poonakar, Wadansamba, Manasagaeam, Kartika amba, Peria kichili, Pasudhandu, Rangoon samba, Pottiatragada, Kavanguri Poothala, Kavanguri Poothala, Erava pandy, Kajal champa, Chhota atte (S), Thula ate, Basmati(s), Pusa Basmati (s), Tapre, Kamla, Joda, Bella, Sarathi, Sonaphal, Nakeswar, Rampal, Matri, Gujuri Samba, Karjat-8, Khudia, Mahsura, Bombay Mahasuri, Suruti, Saat Akra, Indrayani, Takur bug, Kali khasa (s), Ghigaj, Zilong, Kauli, Admachikam, Beti, Kalabiron, Maichakca, Mamichapa, Biron, Bini, Nagra, Red Binni, Black Binni, Maime Kasana, Hari narayan, Gujuri, Gujuri, Rachki, Lachki, Gujarat-70, Sonam (s), Gujrat 70–1, Jaya, Gujarat-4, Gujarat-3, Karchat, Gujarat-11, Akada, Bodthadia(s), Basmati (s), Kolam, Dabol, Gorakhnath-509, and GR-102

### Genetic diversity of blast resistant *R* genes

Functional/ linked markers for twenty four genes (twenty three cloned genes) selected for the present study were distributed over the eight chromosomes (Fig 2). The gene frequency of the twenty four blast *R* genes varied from 8.69 to 100%. The number of positive alleles of *R* genes varied from 5 to 19 in the landraces. Among the studied landraces, Biran from Tripura was found to contain highest number of the *R* genes (19) with positive allele. Three landraces (1.86%), showed positive loci for eighteen *R* genes, seven (4.34%) for seventeen *R* genes, ten (6.21%) for sixteen *R* genes, nineteen (11.80%) for fifteen *R* genes, twenty three (14.28%) for fourteen *R* genes, twenty four (14.90%) for thirteen *R* genes, twenty eight (17.39%) for twelve *R* genes, fourteen (8.69%) for both eleven and ten *R* genes, thirteen (8.07%) for nine *R* genes, four (2.48%) for eight *R* genes, three (1.86%) for seven *R* genes and one landrace (Jaya) from Gujarat contained positive allele for only five *R* genes, respectively (S1 Table).

The presence of *Pish* gene was estimated by visualization of PCR product with the linked markers, RM6648 and RM5811. The average gene frequency was found to be 10.55% with a range from 8.69% (RM5811) to 12.42% (RM6648). The estimation of *Pit R* gene localized on chromosome 1 was determined through visualization of the presence of 733 bp and 530 bp amplicons corresponding to the tk59–1 and tk59–2 markers, respectively. Ninety seven landraces were found to be positive for a *Pit* gene with 60.24% gene frequency. The SSR markers, RM302 and RM212 were used to detect positive alleles of a *Pi37* gene with a gene frequency of 21.11% and 14.90%, respectively for the corresponding markers. The marker, Pb28 could amplify rice blast *Pib* gene with a fragment size of 388 bp. The gene frequency of *Pib* was found to be 26.70%. The recessive blast resistance gene *pi21* located on chromosome 4 was detected by using the InDel marker, pi21_79–3. It was observed that only eight landraces were found positive for the *pi21* gene with a gene frequency of 4.96%. The functional marker, Pi9–i was able to detect the presence of *Pi9* gene and twenty five landraces were found positive with a gene frequency of 15.52%. Similarly, the presence of *Pi2* gene was noticed using the functional marker Pi2–i. Interestingly, only twenty landraces were found positive for the *Pi2* gene with a gene frequency of 12.42%. The functional (dln2) and linked (RM564) markers were used to determine the presence of *Pid2* and *Pi25* genes, respectively. It was revealed from this result that the presence of *Pid2* gene in 151 landraces and *Pi25* gene in 47 landraces with the gene frequency of 98.75% and 29.19%, respectively. The SNP primers, z56592, and zt56591 were used to ascertain the presence of *Piz* and *Piz–t* genes, respectively, which demonstrated the presence of *Piz* in 158 landraces and *Pizt* in 137 landraces. Surprisingly, landraces positive for the *Piz–t* gene were noted to be positive for the *Piz* gene. The rice blast *R* genes, *Pi33* and *Pi36* on chromosome 8 was amplified using the linked marker RM72 and RM5647, respectively which showed the presence of *Pi33* in 15 landraces with a gene frequency of 9.31% and *Pi36* in 99 landraces having a gene frequency of 61.4%. Eighty three landraces were found to harbour the blast resistance gene *Pi5* located on chromosome 9 which was amplified with the marker, 40N23r. Likewise, the presence of *Pi56(t)* gene was observed in seventy two landraces determined by using InDel marker CRG4_2.

The presence of resistance alleles of *Pik* and *Pik–p* genes can be verified by the markers, K39512 and K3957 producing the PCR amplicon size of 112 bp and 148 bp, respectively. Based on these markers, 161 landraces were found to carry *Pik* where as *Pik–p* were distributed in 156 landraces. Uses of the InDel marker, *Pikm*, showed its presence with a fragment size of 619 bp in 97 landraces while the functional marker, *Pikh*, detected this gene in 118 landraces amplifying the fragment size, 216 bp. Further, the broad spectrum blast resistance gene *Pi1* was noticed using two linked markers, RM1233 and RM224 in 61 and 82 landraces respectively, whereas 48 landraces were found positive for both the markers. The PCR based amplification of the neck blast resistance gene *Pb1* showed its presence in seventy nine landraces with a gene frequency of 49.06%. The InDel and STS marker; SNP_3, and Pia–STS were used for the detection of *Pi65(t)* and *Pia* genes, respectively. The present study showed the presence of *Pi65(t)* gene in 89 landraces with a gene frequency of 55.27%, whereas, *Pia* was distributed in 123 landraces with a gene frequency of 76.39%. The presence of broad spectrum *Pita/Pita–2* on chromosome 12 was detected by visualization of 1,042 bp with YL155/87 primer pairs. The *Pita/Pita–2* gene was present in eighty seven landraces showing the gene frequency of 54.0%. In all the PCR amplification, HR-12 was used as a negative control (S2 Table).

The numbers of allele per locus for twenty eight markers varied from 1 to 2 with a mean value of 1.76 whereas the allele frequency ranged from 0.5 to 1.0. The minimum and maximum PIC value for twenty eight markers varied from 0 (K39512) to 0.37 (YL155/YL87, 40N23r, RM26998, RM224, CRG4–2 and SNP_3) with an average value of 0.25. The gene diversity varied from 0 (K39512) to 0.49 (YL155/YL87, 40N23r, RM26998, RM224, CRG4–2 and SNP_3). The PIC value of marker K39512 for *Pik* gene showed the lowest value (0) owing to its monomorphic nature in all the landraces in the present study. In contrast, the markers such as YL155/YL87 (*Pita/Pita–2*), 40N23r (*Pi5*), RM26998 (*Pb1*), RM224 (*Pi1*), CRG4–2 (*Pi56(t)*) and SNP_3 (*Pi65(t*)) were found to be more informative to study the genetic diversity as they showed highest PIC value of 0.3749 (Table 3).

**Table 3:** Estimation of major allele frequency, genotype number, allele number, gene diversity and PIC in landraces.

Marker	Major allele frequency	Genotype number	Allele number	Gene diversity	PIC
Pb28	0.7329	2	2	0.3950	0.3149
Z56592	0.9814	2	2	0.0366	0.0359
Zt56591	0.8509	2	2	0.2537	0.2215
K39512	1.0000	1	1	0.0000	0.0000
K3957	0.9689	2	2	0.0602	0.0584
k2167	0.6025	2	2	0.4790	0.3643
Pikh	0.7329	2	2	0.3915	0.3149
Pi9-i	0.8447	2	2	0.2623	0.2279
Pi2-i	0.8758	2	2	0.2176	0.1939
YL155/YL87	0.5404	2	2	0.4967	0.3734
40N23R	0.5155	2	2	0.4995	0.3748
tk59-1	0.6087	2	2	0.4764	0.3629
tk59-2	0.6025	2	2	0.4790	0.3643
dln2	0.9876	2	2	0.0245	0.0242
RM6648	0.8758	2	2	0.2176	0.1939
RM5811	0.9130	2	2	0.1588	0.1462
RM26998	0.5093	2	2	0.4998	0.3749
RM72	0.9068	2	2	0.1690	0.1547
Pia-STS	0.7640	2	2	0.3606	0.2956
RM1233	0.6211	2	2	0.4707	0.3599
RM224	0.5093	2	2	0.4998	0.3749
pi21_79–3	0.9503	2	2	0.0944	0.0900
CRG4-2	0.5528	2	2	0.4944	0.3722
SNP_3	0.5528	2	2	0.4944	0.3722
RM5647	0.6149	2	2	0.4736	0.3614
RM302	0.7888	2	2	0.3332	0.2777
RM212	0.8509	2	2	0.2537	0.2215
RM564	0.7081	2	2	0.4134	0.3280
**Mean**	**0.7487**	**1.96**	**1.96**	**0.3215**	**0.2555**

### Cluster analysis

**Fig 3:**
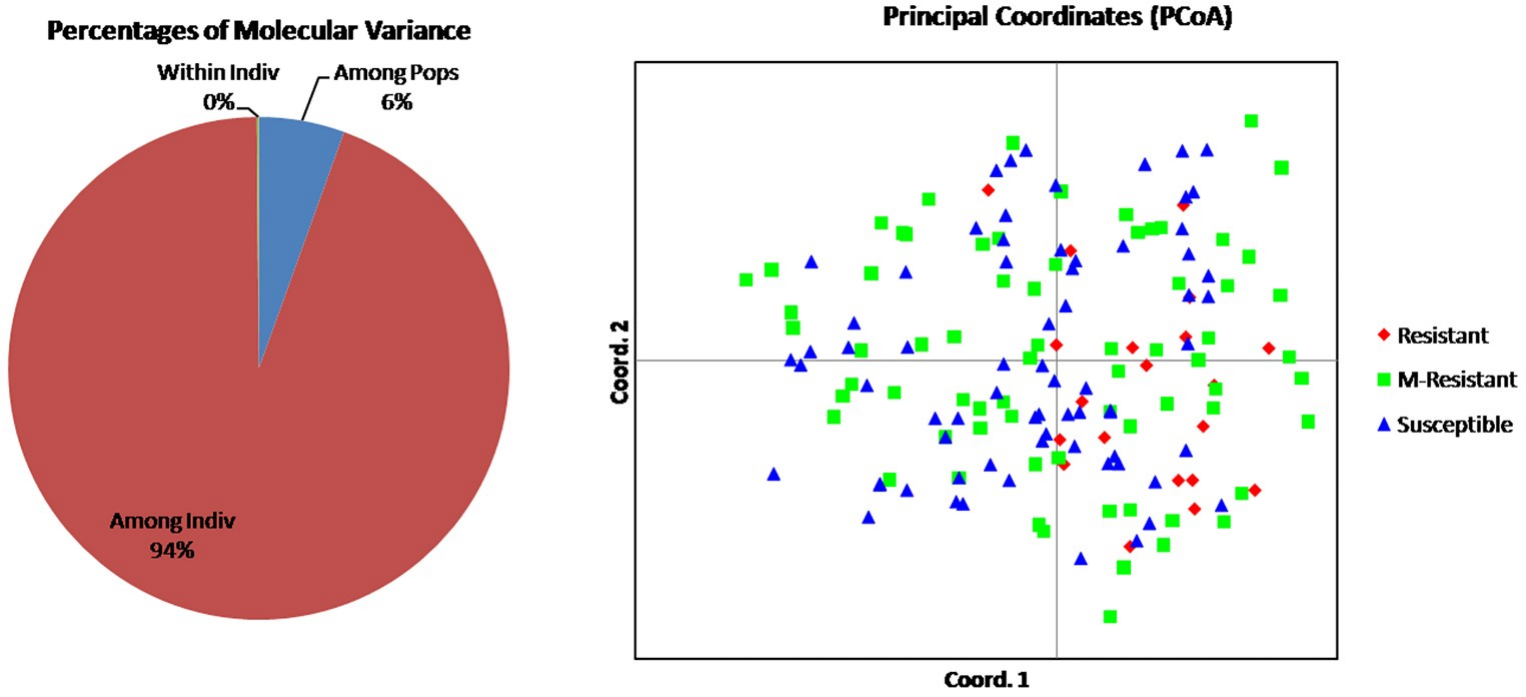
Unrooted neighbor joining tree of 161 rice landraces constructed based on 28 markers data. (Landraces represented in colors corresponding to the sub-population on the basis of population structure (SG1-blue; SG2-red, and admixture-green).

For assessment of the genetic distance, 28 genic/linked markers were used to construct a dendrogram by using the UPGMA method of pooled 28 marker data led to the segregation of 161 landraces into two distinct major groups. The dendrogram was divided in to two major clusters, I and II (Fig 3). Major cluster I consisted of four sub–clusters IA–1, IA–2, IA–3 and IA–4. Sub–cluster IA–1 possessed 26 landraces of which five (19.23%) were resistant genotypes whereas sub–cluster IA2 comprised of two (3.33%) resistant landraces and 3 susceptible genotypes. Sub–cluster IA–3 comprised of thirteen landraces including only one (7.69%) resistant landraces. Similarly, Cluster IA–4 possessed only one (4.55%) resistant landraces along with twenty two genotypes.

Similarly, sub–cluster IB clutched 94 landraces and further grouped into five sub–clusters IB–1, IB–2, IB–3, IB–4 and IB–5. Sub–cluster IB1 contained 30 landraces, with the highest number (six) of resistant landraces. No resistant landraces falls in sub–clusters IB–2 though it consisted of fifteen genotypes. Twenty eight landraces were found in the sub–cluster IB–3 including four (14.29%) resistant landraces. Further, sub–cluster IB–4 and IB–5 consisted of fourteen and seven landraces respectively; none of them were resistant. No resistant landraces were found in sub–cluster IB–4 whereas IB–5 consisted of two (28.57%) resistant landraces. This result shows that the landraces Biran from Tripura state possessing a maximum number of resistance genes (nineteen) falls in the sub–cluster IB–1 whereas a landrace, Jaya of Maharashtra collection carrying the minimum number of resistance genes (five) showing resistant reaction was found in the sub–cluster IA–1; this shows the ability of all 28 markers for discriminating the landraces of maximum and minimum number of resistance genes. Surprisingly, resistant landraces were almost equally distributed in two major clusters. The highly resistant landraces viz: Champe, Badda atte belonged to major cluster II, while landrace Khasa found in major cluster I. This result gives a clear picture that the landraces of the genetically similar forms a group together whereas the landraces belonging to same ecologies did not show any grouping among them.

### Genetic association of marker alleles with blast resistance

Genetic association analyses of the blast resistant genes were calculated using the GLM model of TASSLE5 software to assess the significant association with the blast disease scores. Among twenty eight markers used for association study, only five markers (K3957, Pikh, Pi2–i, RM212 and RM302) for five *R* genes (*Pik–p*, *Pikh*, *Pi2*, *Pi1* and *Pi37*) showed a significant association to the blast disease (Table 4); the associated markers showed a phenotypic variance of 1.4% to 7.6%. Highest phenotypic variance (7.6%) was observed with the marker, K3957 corresponding to *Pik–p* gene. The other marker Pi2–i used for *Pi2* gene represented a phenotypic variance of 3.2%, followed by RM302 marker with phenotypic variance of 2.5%. Surprisingly, other twenty three markers representing nineteen resistance genes did not exhibit any significant association (*P*< 0.1) among them.

**Table 4:** Genetic association of blast resistant genes with rice blast disease in 161 landraces.

S. No.	Gene	Marker	*p*-value	Marker_R^2^
1.	*Pi65*	SNP_3	0.9537	2.13E-05
2.	*Pib*	Pb28	0.3663	0.00514
3.	*Piz*	Z56592	0.42014	0.00409
4.	*Piz-t*	Zt56591	NaN	0
5.	*Pik*	K39512	0.24007	0.00867
6.	*Pik-p*	K3957	3.80E-04**	0.07658
7.	*Pikm*	k2167	0.5327	0.00245
8.	*Pikh*	Pikh	0.06203*	0.02173
9.	*Pi9*	Pi9-i	0.90829	8.37E-05
10.	*Pi2*	Pi2-i	0.02274**	0.0322
11.	*Pita/Pita2*	YL155/YL87	0.64394	0.00135
12.	*Pi5*	40N23R	0.56724	0.00206
13.	*Pit*	tk59-1	0.63302	0.00144
		tk59-2	0.2251	0.00924
14.	*Pid2*	dln2	0.78204	4.83E-04
15.	*Pish*	RM6648	0.85234	2.19E-04
		RM5811	0.58015	0.00193
16.	*Pb1*	RM26998	0.6739	0.00112
17.	*Pi33*	RM72	0.84907	2.28E-04
18.	*Pia*	Pia-STS	0.68348	0.00105
19.	*Pi1*	RM1233	0.13203	0.01421
		RM224	0.91273	7.58E-05
20.	*Pi21*	pi21_79–3	0.92924	4.97E-05
21.	*Pi56(t)*	CRG4-2	0.33877	0.00576
22.	*Pi36*	RM5647	0.35039	0.00549
23.	*Pi37*	RM302	0.04469**	0.02511
		RM212	0.10514*	0.01643
24.	*Pi25*	RM564	0.52978	0.00249

* & ** Significant at *P* value <0.1 and <0.05 respectively

### Estimation of population genetics through AMOVA analysis

**Fig 4:**
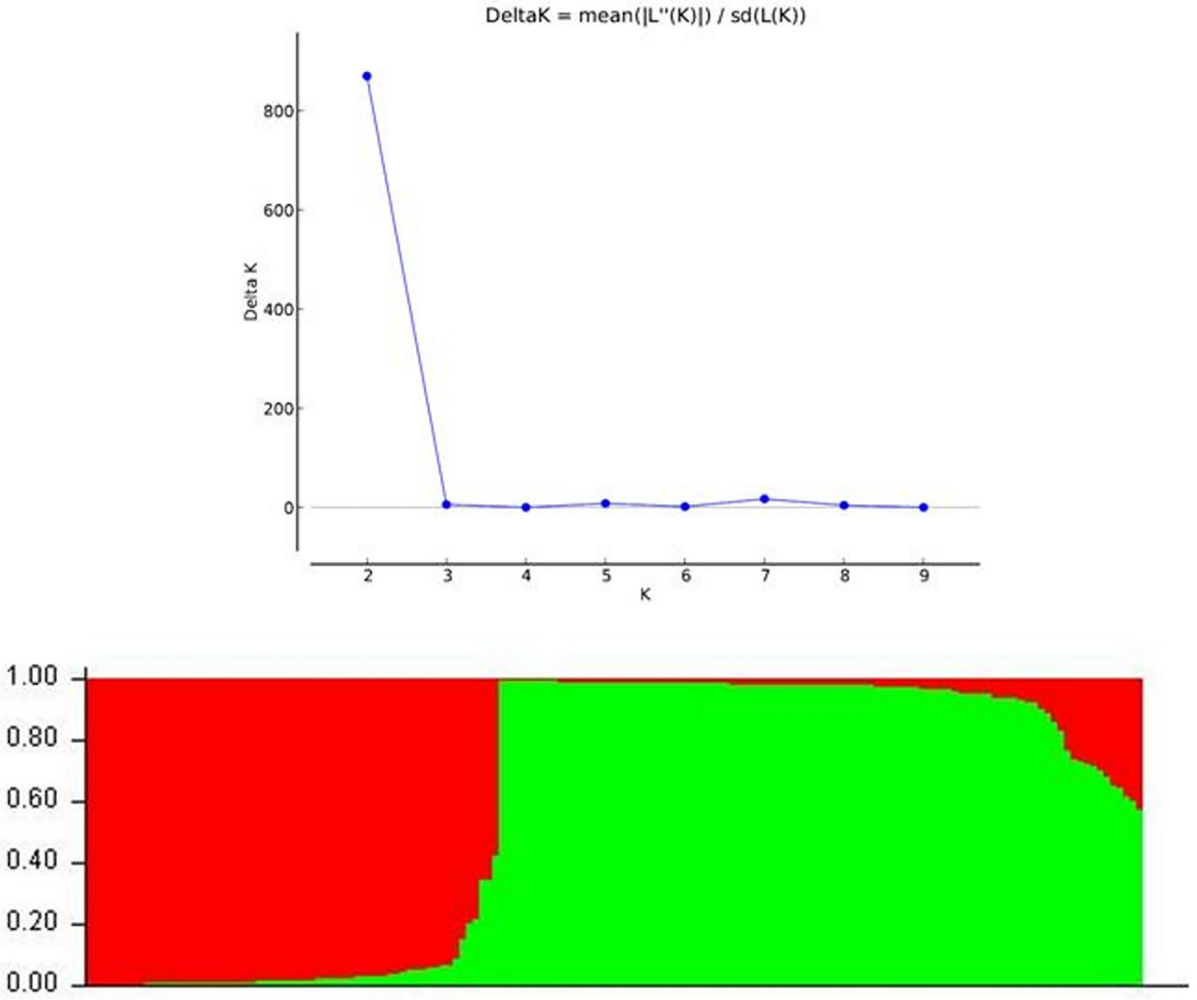
Analysis of molecular variance (AMOVA) and Principal Coordinate Analysis (PCoA) of 161 Indian landraces based on linked/functional markers.

An AMOVA analysis was conducted to assess the existence of genetic diversity within and between the populations. Based on the disease score, 161 landraces were divided into three populations: HR (21), MR (70) and S (70). Further, it was observed that highest diversity (94%) exists within population, whereas least (6%) between population (Table 5; Fig 4). The pair–wise fixation indices (F_ST_) among the populations are listed in the Table 6. The pair wise F_ST_ estimate was highest between the highly resistant and susceptible while it was least between the highly resistant and moderately resistant populations. Based on the estimated value of fixation indices, it is indicated that there is weak population structure and they are not also isolated genetically from each other.

**Table 5:** Analysis of molecular variance (AMOVA).

Source	Df	SS	MS	Est. Var.	% of variation
**Among Pops**	2	67.246	33.623	0.255	6%
**Within population**	158	1382.767	8.752	4.373	94%
**Within individual**	161	1.000	0.006	0.006	0%
**Total**	321	1451.012		4.634	100%

Df: degree of freedom; SS; sum of squares, Est. Var.: Estimated variance

**Table 6:** Pair–wise F_ST_ estimates among the three populations of rice landraces.

Populations	Highly resistant	Moderately resistant	Susceptible
**Highly resistant**	0.000	0.001	0.001
**Moderately resistant**	0.077	0.000	0.001
**Susceptible**	0.124	0.026	0.000

F_ST_ values below diagonal.

The PCoA was conducted to establish the genetic relatedness of 161 landraces based on the 28 markers corresponding to the 24 blast *R* gene data. The PCoA analysis showed a scattered plot where the first two axes explained variations of 15.66% and 9.56%, accounting to total of 25.22% of genetic variation. The scatter plots have also clearly distributed the resistant landraces mostly in second and forth quadrants whereas moderately and susceptible landraces were distributed in all the four quadrants (Fig 4). Similarly, population assignment study using GenAlEx, clearly differentiated the resistant and susceptible landraces however, it was not able to differentiate moderately-resistant and susceptible populations (S2 Fig).

### Population structure analysis

**Fig 5:**
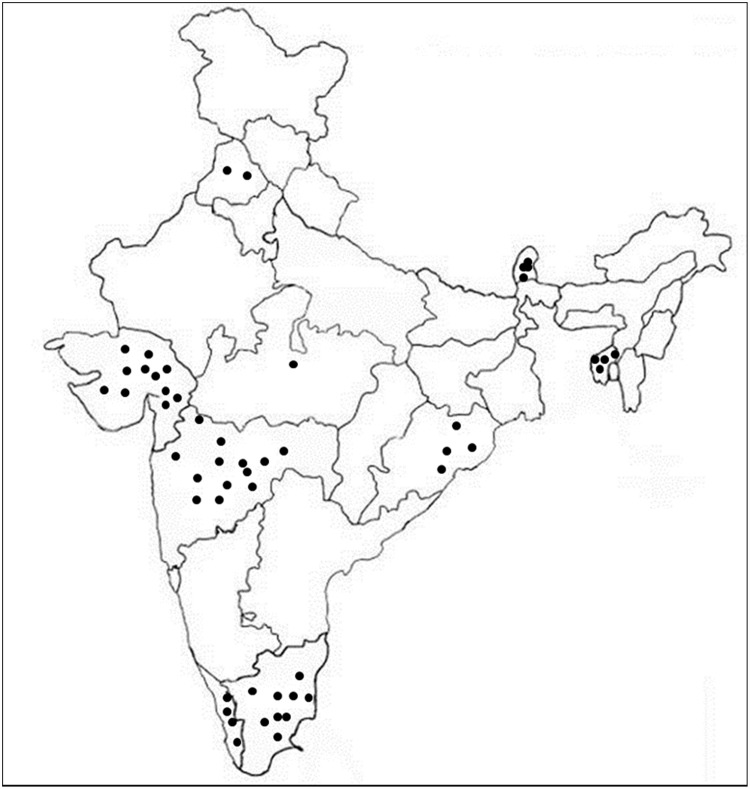
Population structure of Indian landraces based on 28 marker for blast resistance genes.

All the 161 landraces were evaluated for estimation of population structure for blast disease based on 28 markers corresponded to 24 blast resistance genes using Structure software. The peak plateau of adhoc measure ΔK was found to be K = 2 (Fig 5), which indicated that the entire landraces were distributed into two subgroups (SG1 and SG2). Based on the ancestry threshold of >60%, all landraces were classified into two subgroups with two admixture (S3 Table). The SG1 was the small group consisting 62 landraces (38.50%) of which 35 and 27 were resistant and susceptible, respectively. The SG1 contained nine highly resistant landraces. In contrast, the SG2 included 97 landraces (60.24%) of which 54 and 43 landraces were found to be resistant and susceptible, respectively. There were more numbers of resistant landraces (54) in the SG2 cluster as compared to SG1. Among three most resistant landraces, two (Champe and Bada atte) were belonged to SG2 cluster. In SG2, there were only 12 genotypes which were found to be highly resistant (HR). If the classification is based on the criteria of highly resistant (HR; score 0–3) and susceptible scores (MR and SS; score 4–9), then there are only nine HR landraces (14.51%) in SG1 as compared to twelve HR genotypes (12.37%) in SG2.

## Discussion

The widespread use of high–yielding varieties has significantly lowered the genetic base of plant breeding material of agriculturally important food crops, which restricts their future improvements [32, 33]. Cultivation of genetically uniform varieties over large scale imposed high selection pressure on the pathogen populations which leads the cultivars highly prone to biotic stress. Changing climate and the emergence of new virulent races imposed a continuous threat to the rice production and global food security. Accordingly, a protection measure necessitates constant progress to keep pace with the evolving pathogen [34]. This needs the identification of new resistance genes and alleles from landraces and wild relatives. The genetic diversity of the majority crop plants is being stored in the form of germplasm/accessions in the gene banks. However, the genotypic diversity of most of the accessions has not been fully explored and understood [35]. In this study, we investigated the genetic diversity of geographically diverse Indian landraces which are unique, unexplored and untapped germplasm for blast resistance genes that originated from nine major rice growing states of India with diverse ecology using major blast resistance genes.

A few landraces like Jaya possessed only five *R* genes and Suruti, Karjat–2 and Gujari contained seven *R* genes out of the 24 *R* genes analyzed which exhibited resistant reaction. Interestingly, most of the landraces belonged to upland rice showed the less number of *R* genes. Some landraces carrying only functional four-five *R* genes but still showed resistance reaction against the blast pathogen which might be due to presence of novel *R*–gene(s) or the combination of major *R* gene and major quantitative trait loci or minor gene interactions [36].

On the other hand, some landraces such as Erava pandy, Basmati(s), Maichakca, Gujuri, Kajal champa, Maichakca and Red binni found to be carrying fourteen or more *R* genes intriguingly showed susceptible reaction (Score 7). Similarly, Biran landrace from Tripura state was found positive for nineteen *R* genes and displayed moderate resistance which corroborated the findings of Yadav et al. [9], where few cultivars showed susceptible reaction despite having maximum number of resistance genes. Similar results have been reported in rice blast disease [9,36] which might be explained by the type of allele of the *R* genes presence in these landraces or resistant breading down due to mutations occurred in the *R* genes or evolution of new pathogen races.

The availability of linked molecular markers for detection of *R* genes for blast resistance can be used in diverse elite genetic resources [37,38,39,40,41]. Similarly, functional markers for blast resistance genes have been widely used for mining of *R* genes in elite germplasm to know the presence of blast resistance genes [39,40,42,43,44]. In the present study, uses of 28 genic/linked markers have shown the occurrence of variation in genetic frequencies of 24 blast resistance genes from 8.69 to 100% and number of *R* genes from 5 to 19. Likewise, the genetic frequency of the twelve major blast resistance genes varied from 0 to 100% in NRVs and 6 to 97% in North East and Eastern germplasm for nine blast resistance genes [9,45].

The presence of *Pish* gene was detected in fourteen (12.42%) and twenty (8.69%) landraces using linked marker RM58118 and RM664, respectively. Similarly, *Pit* gene was reported in the ninety seven landraces with a gene frequency of 60.24%. A similar study was carried out by Yang et al. [46] which showed the presence of the *Pit* gene in 14 (3.9%) among 358 rice accessions. The result of *Pi37* gene showed its gene frequency of 21.11% and 14.90%, respectively corresponding to the linked markers, RM302 and RM212. Interestingly, *pi21* gene was found positive only in eight landraces exhibiting the gene frequency of 4.96%. The *Pib* gene was found in 43 landraces which was also detected in NRRI released varieties, North East and Eastern germplasm and Manipur rice accessions [9,45,47]). The positive loci of *Pi9* gene was observed in twenty five (15.52%) landraces. Similarly, it was detected in 15 NRVs, 2 North East and Eastern Indian rice [9,45]. Likewise, the *Pi2* gene was detected in twenty (12.42%) landraces whereas, it was observed in 14 accessions of the tested 358 accessions [46]. The *Pid2* and *Pi25* genes showed its presence in 151 (98.75%) and 47 (29.19%) landraces, respectively. The presence of *Piz* and *Piz–t* genes was demonstrated in 158 and 137 landraces, respectively, which is concurrent with the previous reports where these two genes conferred partial resistance to the tested entries [9,45].

The rice blast *R* gene, *Pi33* and *Pi36* showed its presence in 15 (9.31%) and 99 (61.4%) landraces, respectively. Similarly, *Pi33* gene was observed in 77 (40.10%) of tested accessions [48]. Seventy two (62%) landraces were found to harbour *Pi56(t)* gene. The *Pi5* gene was detected in eighty three (51.55%) landraces whereas previous studies showed its presence in 60 landraces from Karnataka, 4 from Manipur and 26 NRVs [36,47,9]. The multiple gene complex loci is provided by the *Pik* locus on chromosome 11 possessing at least five genes, *Pik*, *Pikm*, *Pikh*, *Pikp*, and *Piks* [38]. The *Pik*, *Pik–p* and *Pikm* genes appeared in 161 (100%), 156 (96.89%) and 97 (60.24%) landraces, respectively. Likewise, these genes were found to be present in maximum frequency in the NRVs [9]. Interestingly, the *Pik* gene was detected in the majority of the accessions tested [45,9]. The broad spectrum resistance gene *Pikh* isolated from Tetep variety was scored in one hundred eighteen (73.29%) landraces. Similarly, this gene was detected in fifty–six (70%) NRVs and in another study it was observed in 18 and 52 accessions [9,45,48]. The broad spectrum *Pi1* gene was scored in sixty one (RM1233) and eighty two (RM224) landraces. In another study, it was observed in 39 landraces and 20 NRVs [36,9]. This study showed the presence of *Pb1*, *Pi65(t)* and *Pia* genes in seventy nine (49.06%), eighty (55.27%) and one hundred twenty three (76.39%) landraces, respectively. The broad spectrum *Pita/Pita–2* was present in eighty seven (54%) landraces. Similarly, other workers reported its presence in 32.50%, 19.29%, 6.25% and 27% accessions [9,48,45,49]. Intriguingly, in the present study there was no strong relationship between known *R* genes and disease reaction.

In the current study, cluster analysis categorized the 161 landraces into two major sub–clusters. It was observed that the genetically similar landraces of each major cluster were distinguished with landraces of diverse ecologies. Population structure analysis based on twenty eight markers corresponding to twenty four *R* genes could differentiate the entire landraces into two sub–populations (SG1 and SG2) with only two admixtures. The landraces belonged to the SG1 and SG2 were observed to be concomitant with the two major clusters IB and IA, respectively. Interestingly, the cluster analysis could discriminate all the resistant and susceptible landraces from 161 landraces tested which is similar to the study of Yadav et al. [9]. The PCoA analysis, partitioned the resistant landraces in two quadrants whereas, moderately resistant and susceptible landraces were distributed in all four quadrants. Population assignment evidently differentiated the resistant and susceptible landraces but did not distinguish between moderately resistant and susceptible landraces.

An AMOVA analysis indicated the existence of maximum diversity (94%) within population and minimum (6%) diversity between populations which is similar to NRRI released varieties [9]. Similarly, highest fixation index values (Fst) were observed between highly resistant and susceptible landraces and least for resistant and moderately resistant populations. The observed fixation indices represent that overall population structure is a weak, and genetically related to each other.

Association mapping is an important strategy for the discovery of novel genes for important traits and identification of potential donor for their utilization in rice improvement [50]. The present study showed the association of five markers; K3957, Pikh, Pi2–i, RM212 and RM302 associated to five *R* genes such as *Pik–p*, *Pikh*, *Pi2*, *Pi1* and *Pi37*. This result could exhibit a robust marker by utilization of associated markers to assess the genetic diversity of rice blast resistance genes in diverse germplasm. However, our study did not described the complete association between the phenotypic reaction and resistance gene(s) which could be explained by addition of more blast *R* gene markers or these landraces could to be tested for identification of novel *R* genes/alleles or QTLs that can be utilized in rice breeding programs.

## Conclusion

The phenotypic screening and molecular characterization of blast resistance genes will help in identification of potential germplasm for leaf blast. Our results offered an outline of the genotypic diversity of Indian landraces representing nine major rice growing states of India with diverse ecologies. Besides, the precise screening of leaf blast for the identification of resistance genes in landraces along with the identified associated marker could be used for the selection of parental materials and the development of resistant breeding lines. Identification of resistant landraces from diverse ecologies will help in better utilization of these landraces as a donor for improvement of existing varieties with blast resistance. Potential landraces for blast resistance could be utilized for mapping to discover novel blast *R* gene(s) and identification of potential donors for their use in rice breeding. Additionally, the genotyping and phenotyping data of 161 landraces generated in this study could be quiet useful to identify the novel blast *R* gene(s) using association mapping in a precise way.

## Supporting information

S1 Fig Reaction of landraces to leaf blast disease in the uniform blast nursery. (TIF)

S2 Fig Population assignment of landraces described by GenAlEx charts representing the log likelihood data of landrace using disease reaction: a) Moderately resistant and resistant and populations b) Susceptible and resistant populations c) Susceptible and moderately resistant populations. (TIF)

S1 Table Details of landraces used in the current study. (XLSX)

S2 Table Genotyping of 161 landraces using markers associated with blast resistance genes, associated gene frequency and their phenotypic reaction to leaf blast in uniform blast nursery. (XLSX)

S3 Table Population structure group of 161 landraces based on inferred ancestry values. (XLSX)
