# Correction: Blast resistance in Indian rice landraces: Genetic dissection by gene specific markers

**DOI:** 10.1371/journal.pone.0213566

**Published:** 2019-03-05

**Authors:** Manoj Kumar Yadav, S. Aravindan, Umakanta Ngangkham, S. Raghu, S. R. Prabhukarthikeyan, U. Keerthana, B. C. Marndi, Totan Adak, Susmita Munda, Rupesh Deshmukh, D. Pramesh, Sanghamitra Samantaray, P. C. Rath

The images for Figs 1–5 are incorrectly switched. The image that appears as Fig 5 should be Fig 1. The image that appears as Fig 1 should be Fig 2. The image that appears as Fig 2 should be Fig 3. The image that appears as Fig 3 should be Fig 4. The image that appears as Fig 4 should be Fig 5. The figure captions appear in the correct order. Please view the correct figures here.

**Fig 1:**
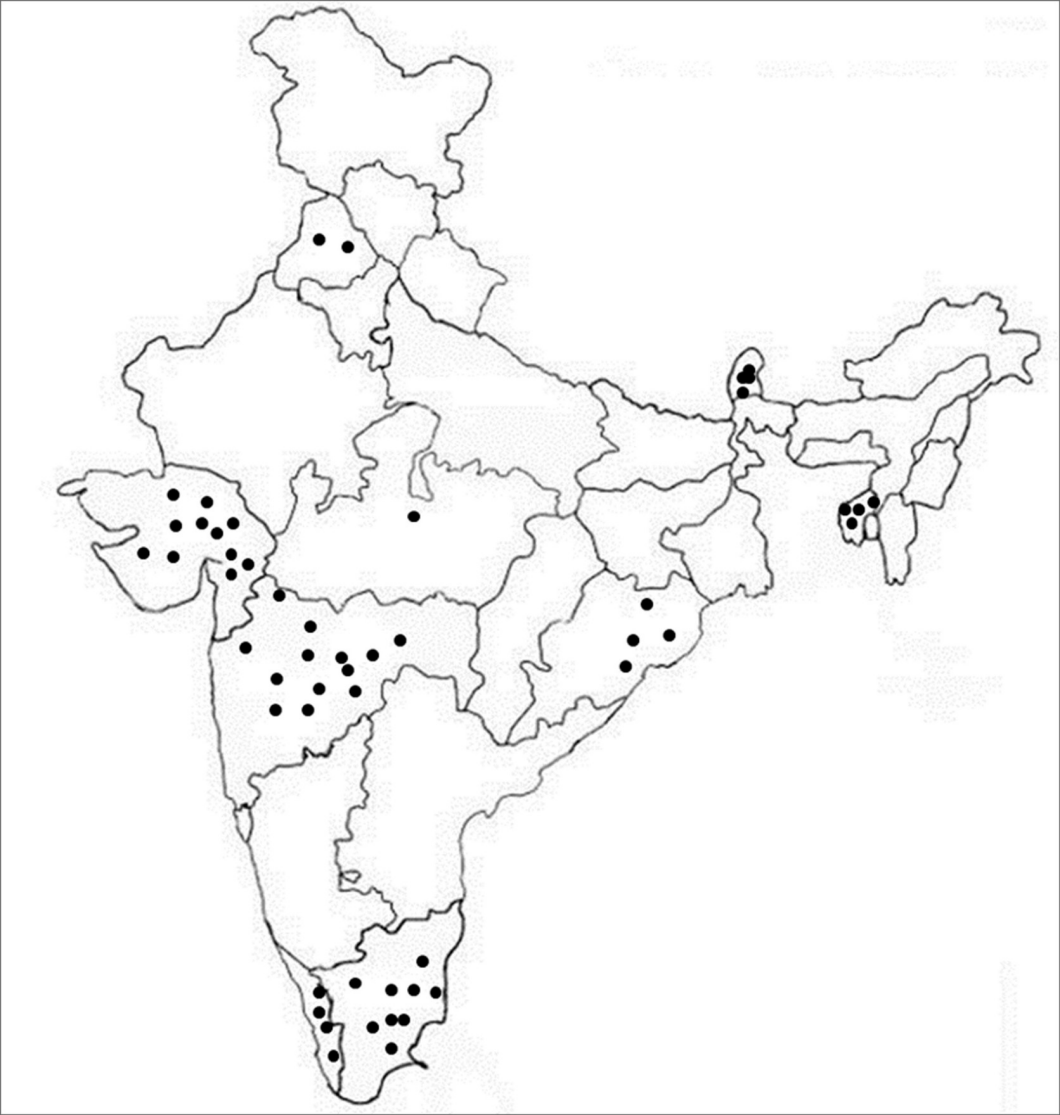
Diagrammatic representation of rice landraces belonged to different states of India.

**Fig 2:**
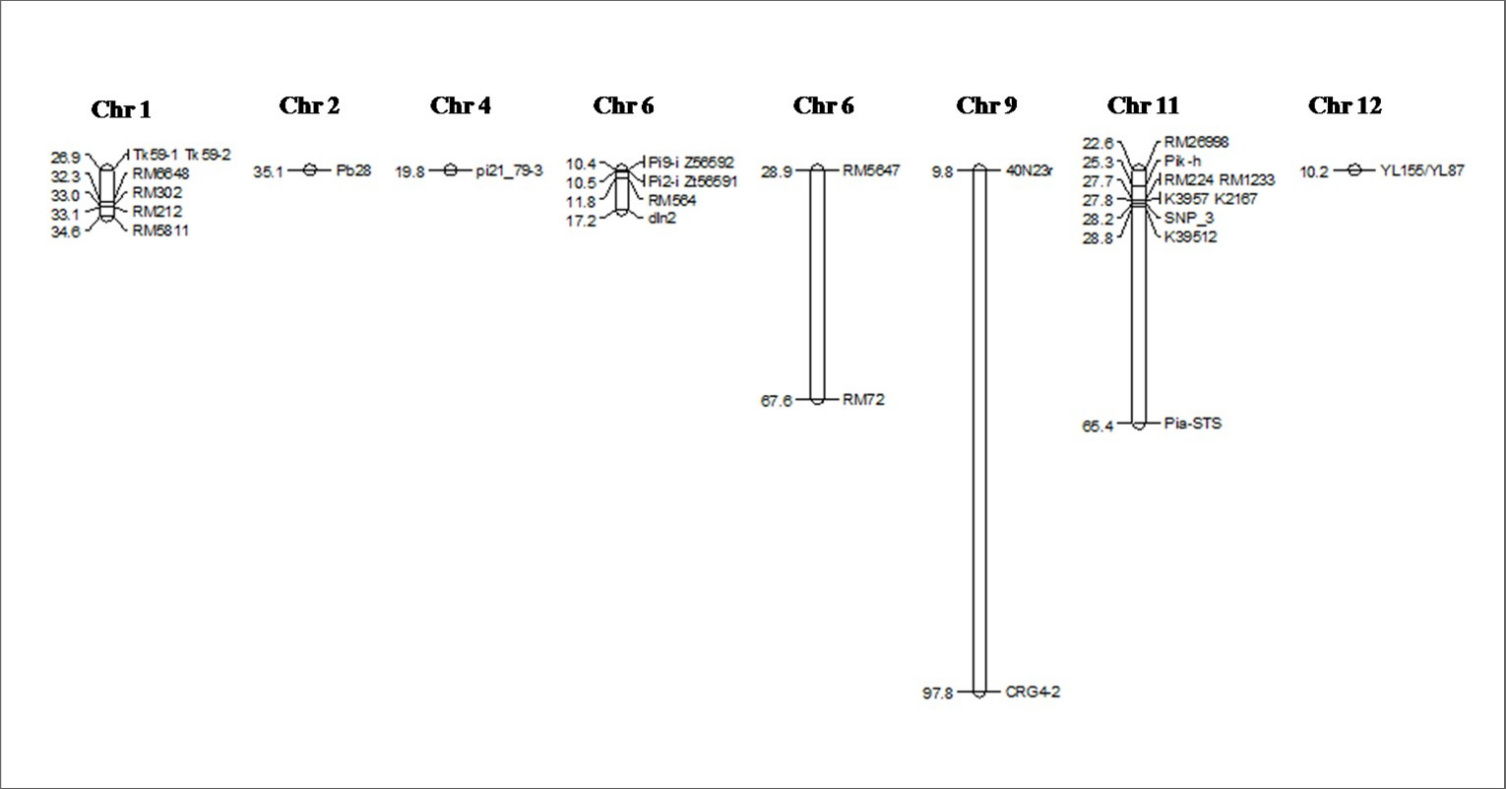
Graphical representation of blast R genes distribution on chromosomes showing the physical location. The names of the markers are given on the right side and the physical positions on the left side of the map.

**Fig 3:**
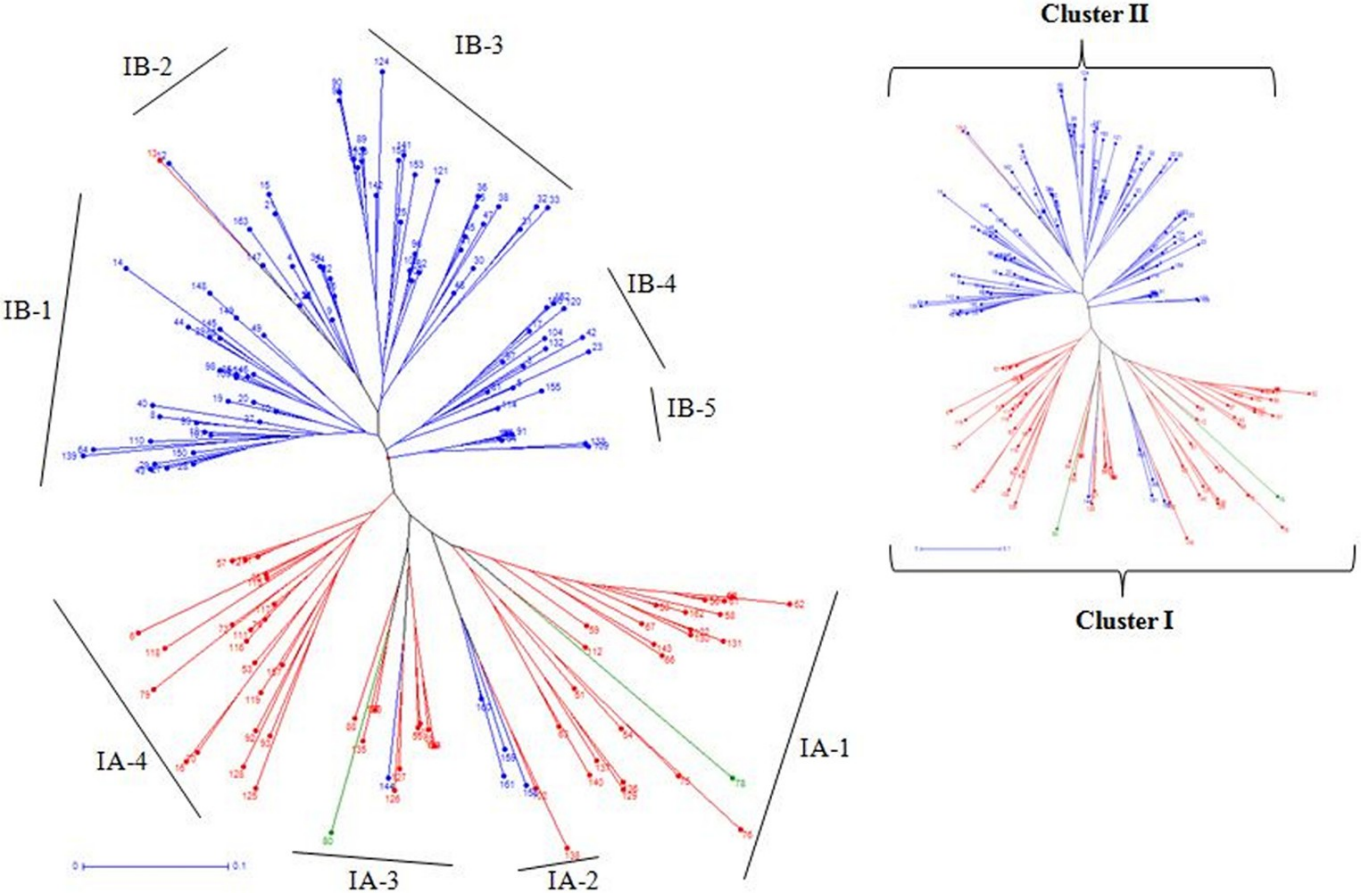
Unrooted neighbor joining tree of 161 rice landraces constructed based on 28 markers data. (Landraces represented in colors corresponding to the sub-population on the basis of population structure (SG1-blue; SG2-red, and admixture-green).

**Fig 4:**
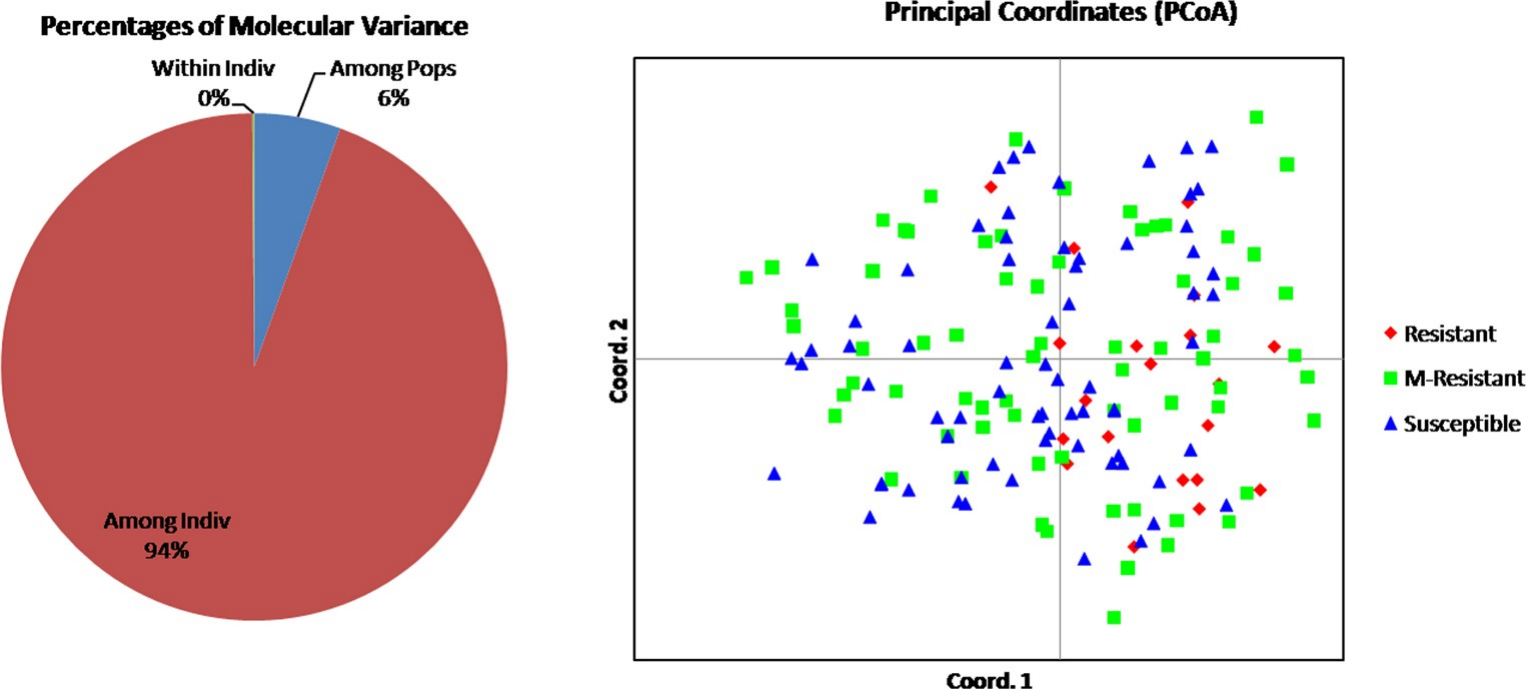
Analysis of molecular variance (AMOVA) and Principal Coordinate Analysis (PCoA) of 161 Indian landraces based on linked/functional markers.

**Fig 5:**
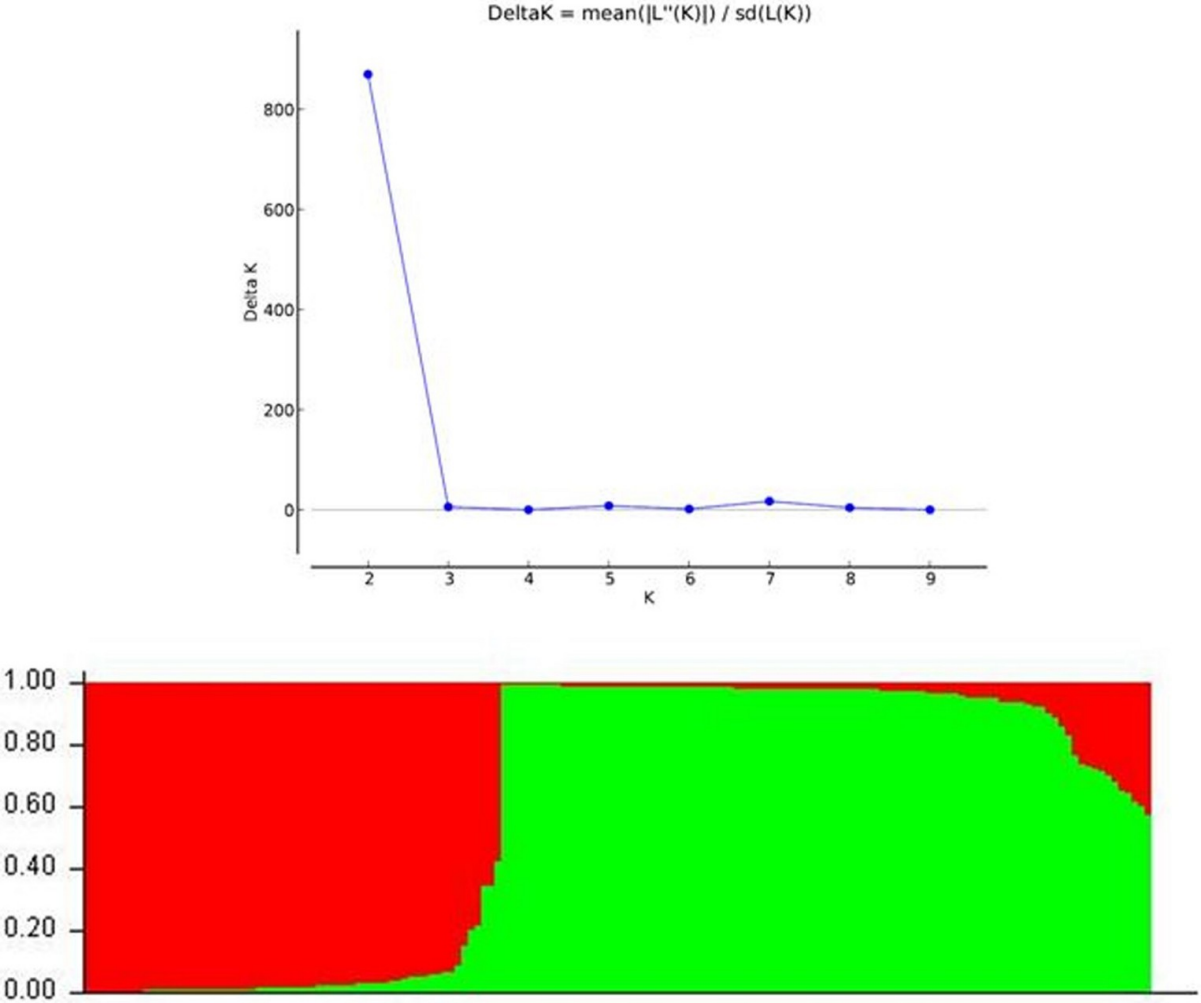
Population structure of Indian landraces based on 28 marker for blast resistance genes.
