# Supplementary figures and images for: Blast resistance in Indian rice landraces: Genetic dissection by gene specific markers
Source: PLoS One. 2019 Jan 23;14(1):e0211061. doi: 10.1371/journal.pone.0211061 (PMC6343911; doi:10.1371/journal.pone.0211061)

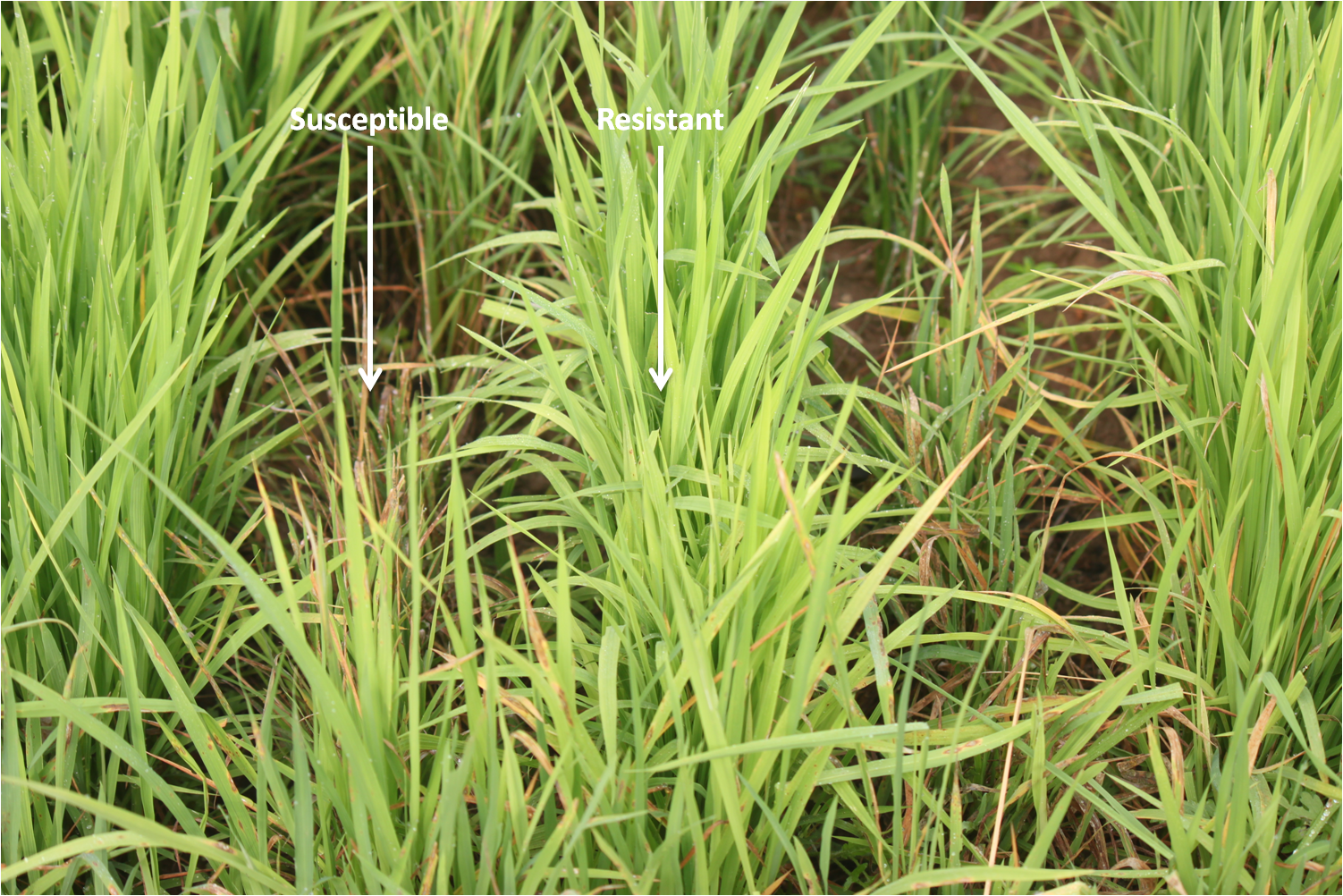

Supplement: S1 Fig — (TIF) [file pone.0211061.s001.tif]

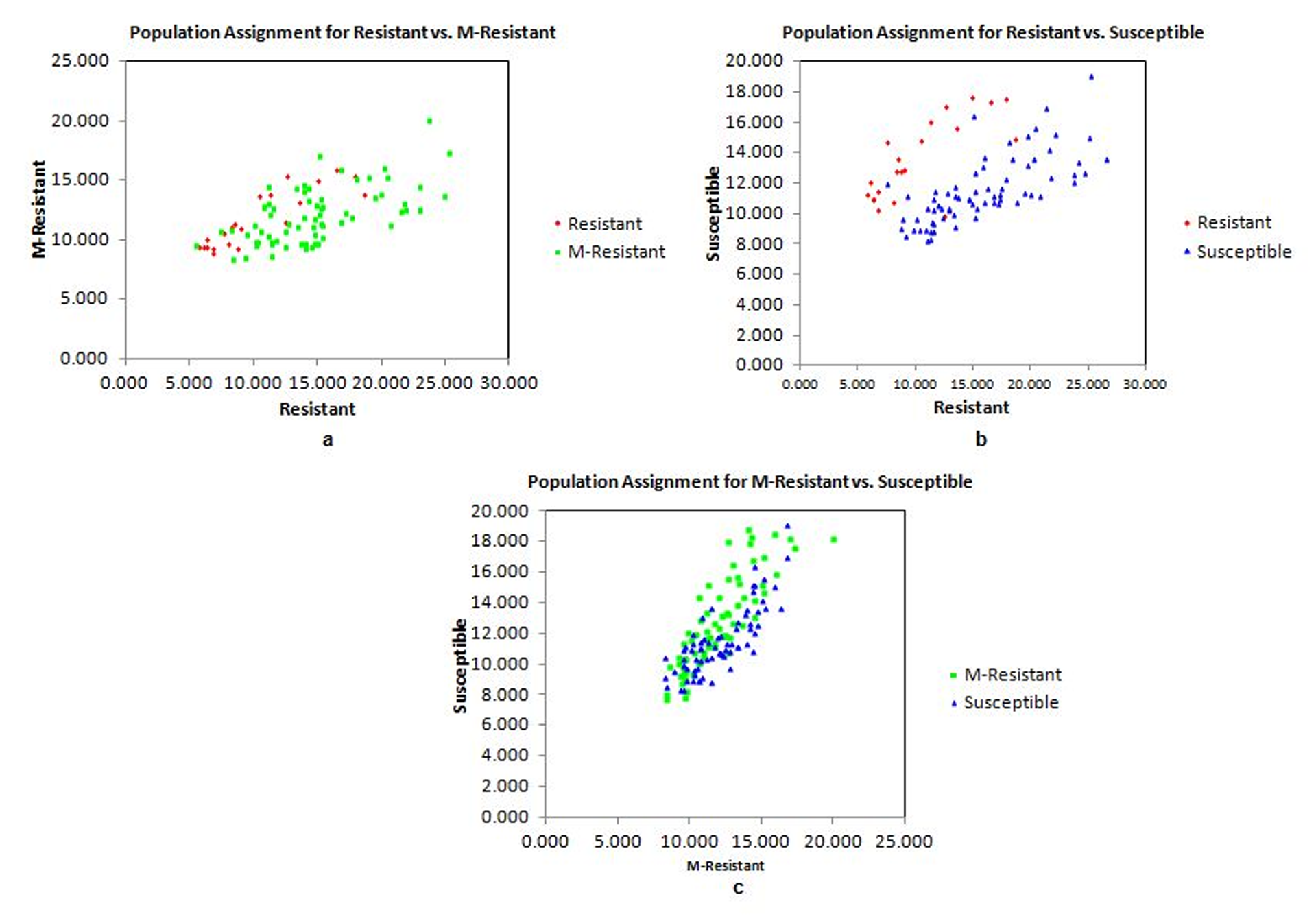

Supplement: S2 Fig — (TIF) [file pone.0211061.s002.tif]
